# Optimized Transcriptional Signature for Evaluation of MEK/ERK Pathway Baseline Activity and Long-Term Modulations in Ovarian Cancer

**DOI:** 10.3390/ijms232113365

**Published:** 2022-11-01

**Authors:** Mikhail S. Chesnokov, Anil Yadav, Ilana Chefetz

**Affiliations:** 1 The Hormel Institute, University of Minnesota, Austin, MN 55912, USA; 2 Masonic Cancer Center, Minneapolis, MN 55455, USA; 3 Stem Cell Institute, Minneapolis, MN 55455, USA

**Keywords:** ovarian cancer, chemoresistance, MAPK

## Abstract

Ovarian cancer is the most aggressive and lethal of all gynecologic malignancies. The high activity of the MEK/ERK signaling pathway is tightly associated with tumor growth, high recurrence rate, and treatment resistance. Several transcriptional signatures were proposed recently for evaluation of MEK/ERK activity in tumor tissue. In the present study, we validated the performance of a robust multi-cancer MPAS 10-gene signature in various experimental models and publicly available sets of ovarian cancer samples. Expression of four MPAS genes (*PHLDA1*, *DUSP4*, *EPHA2*, and *SPRY4*) displayed reproducible responses to MEK/ERK activity modulations across several experimental models *in vitro* and *in vivo*. Levels of *PHLDA1*, *DUSP4*, and *EPHA2* expression were also significantly associated with baseline levels of MEK/ERK pathway activity in multiple human ovarian cancer cell lines and ovarian cancer patient samples available from the TCGA database. Initial platinum therapy resistance and advanced age at diagnosis were independently associated with poor overall patient survival. Taken together, our results demonstrate that the performance of transcriptional signatures is significantly affected by tissue specificity and aspects of particular experimental models. We therefore propose that gene expression signatures derived from comprehensive multi-cancer studies should be always validated for each cancer type.

## 1. Introduction

Ovarian cancer is the fifth leading cause of death among all cancers in women, with a mortality rate exceeding 60% [1]. Most ovarian cancers are high-grade serous ovarian carcinomas (HGSOC), an epithelial tumor subtype that is distinguished by its high aggressiveness, lack of approaches for early diagnosis, and therefore poor prognosis [2,3,4]. Current HGSOC treatment strategies revolve around the administration of platinum-based chemotherapeutic agents (cisplatin, carboplatin, etc.) that initially are effective in most cases. Subsequent relapse and acquired treatment resistance remain a major challenge [5,6,7,8]. Other drugs affecting cancer cells in either a systemic (taxanes, doxorubicin) or targeted way (inhibitors of poly-(ADP-ribose)-polymerase, vascular endothelial growth factor, topoisomerase) are used to bypass platinum resistance, but eventually ovarian tumors develop multi-drug resistance through various molecular mechanisms [9]. It is therefore very important to establish new treatment approaches that would target either known resistance-associated genes or new pro-oncogenic regulatory pathways.

While many regulatory circuits involved in ovarian cancer drug resistance were known for a long time [9], new potentially targetable pathways are being revealed based on genomic, epigenomic, and transcriptomic data analysis [10]. One of them is the mitogen-activated protein kinase 1/2 (MEK1/2)/extracellular signal-regulated kinase 1/2 (ERK1/2) branch of the mitogen-activated protein kinase (MAPK) pathway, a crucial proliferation and survival regulator in most cells, especially malignant ones [11,12]. While the MEK/ERK pathway in tumors has been thoroughly studied [12,13], its role in ovarian cancer is just now being assessed. Hyperactivation of MEK1/2 and ERK1/2 is often caused by activating mutations in Kirsten rat sarcoma viral oncogene homolog (*KRAS*) and V-Raf murine sarcoma viral oncogene homolog B (*BRAF*) genes, and therefore was considered to be confined to low-grade ovarian tumors only [14,15]. However, recent analyses of accumulated genomic data revealed that HGSOC, while rarely harboring *KRAS*/*BRAF* mutations, still often displays amplifications and overexpression of MEK1/2 pathway elements [16]. High MEK1/2 activity was experimentally confirmed in HGSOC cell lines and clinical samples and identified as a negative prognostic factor [17,18]. MEK1/2 activation occurs in response to cisplatin and may render tumors resistant to platinum treatment [18,19,20]. Moreover, the MEK1/2 pathway affects the subpopulation of chemoresistant ovarian cancer stem-like cells (CSCs) defined by high aldehyde dehydrogenase 1 (ALDH1) levels [18,21,22,23]. Taken together, these data clearly support the importance of the MEK1/2-ERK1/2 pathway in HGSOC and its high potential as a therapeutic target, since it is both a regulator of HGSOC development and a pathway involved in drug resistance.

The direct detection of total or active (e.g., phosphorylated) protein is a reliable way to evaluate signaling pathway activity, but is challenging to perform in small samples (for example, obtained via fine-needle aspiration). Moreover, the detectable level of regulatory protein itself, its real activity level, and its effects on downstream targets are not always tightly connected [24,25]. For the MEK1/2 pathway, the response to MEK inhibitors cannot be reliably predicted based on the evaluation of phosphorylated ERK1/2 alone [26]. It is therefore useful to define specific signatures of genes/proteins regulated by pathways of interest as more robust and reliable indicators of signaling pathway activity than single-molecule biomarkers.

An overwhelming number of genes associated with KRAS/BRAF/MEK/ERK activity have been reported in the literature (summarized in [26]). Attempts to discern a simpler and more relevant MEK1/2-specific signature have been made recently. Two 18-gene and 13-gene signatures predicted sensitivity to the selective MEK1/2 inhibitor selumetinib in a multi-type cancer cell line panel [26] and were independently confirmed in a set of gastric cancer cell lines [27]. A set of 24 genes encoding MEK1/2 pathway proteins displayed significant prognostic potential and associations with metabolic aberrations in hepatocellular carcinoma [28]. In addition, a 20-gene signature that predicted survival was identified in cisplatin-sensitive HGSOC based on proteomic and transcriptomic data [17].

Recently, a robust 10-gene signature, the MAPK Pathway Activity Score (MPAS), was described as a potent tool to predict the sensitivity of multiple different tumor cells to MEK inhibition [29]. Instead of being identified through analysis of large transcriptomic datasets, this MEK-specific signature was built based on the manual selection of direct MEK/ERK targets reported in multiple types of cancer. High MPAS was associated with higher sensitivity to MEK1/2 inhibition by cobimetinib in multiple malignant cell lines, increased response to the BRAF inhibitor vemurafenib in BRAF^V600^-positive melanoma patients, and negative survival prognosis. Earlier, we successfully utilized MPAS genes to confirm MEK/ERK pathway inhibition by trametinib in HGSOC cell lines and xenografts [18]. In the present study, we aimed to further refine the MPAS gene signature and improve its efficiency in HGSOC by functionally linking MEK/ERK activity to background levels of MPAS genes and changes, using HGSOC cell lines and patient samples from the Cancer Genome Atlas database.

## 2. Results

### 2.1. PHLDA1, SPRY4, EPHA2, and DUSP4 Are the Best MEK/ERK Responders among Ten MPAS Genes

The expression of the MPAS gene signature is associated with MEK/ERK pathway activity across various tumors [29]. Therefore, we used it to confirm MEK/ERK inhibition by trametinib in ovarian cancer [18]. Most MPAS genes displayed prominent downregulation in response to trametinib, but two genes (*DUSP6* and *CCND1*) displayed inconsistent results, and *EPHA4* expression levels were below reliable levels of detection. The original MPAS study demonstrated that the contribution of single genes to the final MPAS signature can be tissue-specific, so we optimized this approach for ovarian cancer samples.

**Figure 1:**
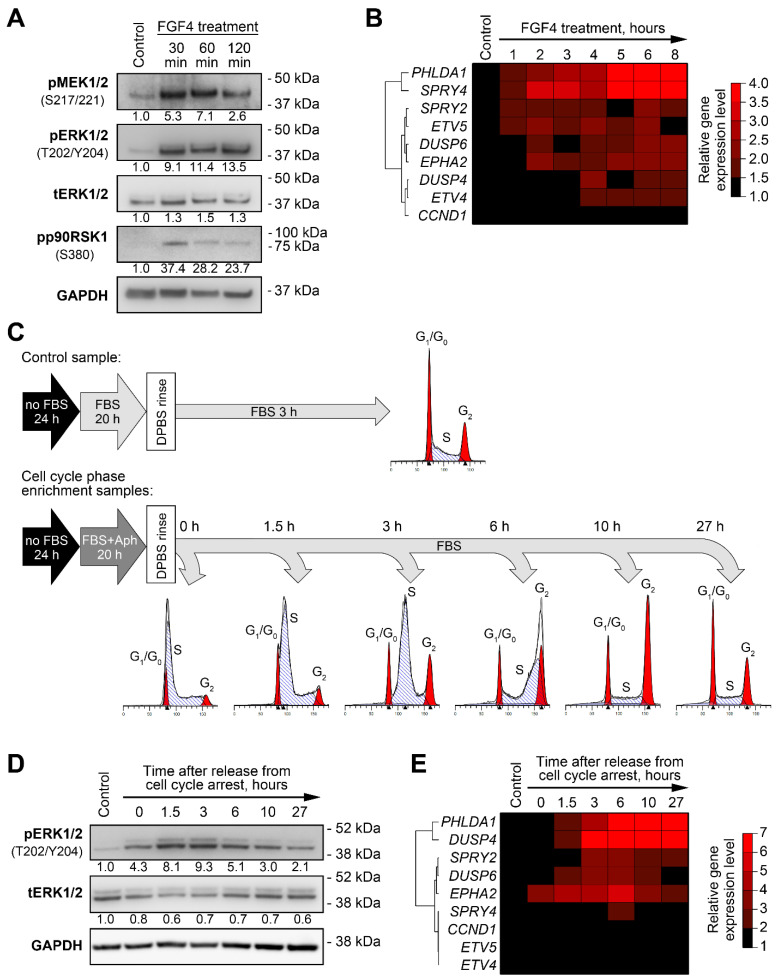
Changes in expression levels of MPAS genes induced by MEK/ERK pathway activity modulation in PEO4 cells. (**A**) Immunoblotting analysis of phosphorylation of MEK/ERK pathway components in response to treatment with FGF4 (100 ng/mL). Numbers under the bands represent relative intensity normalized to GAPDH levels and control samples. (**B**) Changes in expression of MPAS genes in FGF4-treated cells. Heatmap represents gene expression levels normalized to control samples. Unsupervised hierarchical clustering was performed using 1-minus Spearman’s rank correlation metrics. (**C**) Experimental design for enrichment of cultured cells in different phases of cell cycle (see Methods for details). Aph—aphidicolin. (**D**) Immunoblotting analysis of ERK1/2 phosphorylation changes during cell cycle progression in synchronized cells. Numbers under the bands represent relative intensity normalized to GAPDH levels and control samples. (**E**) Changes in expression of MPAS genes during cell cycle progression. Heatmap represents gene expression levels normalized to control samples. Unsupervised hierarchical clustering was performed using 1-minus Spearman’s rank correlation metrics.

From the functional point of view, the best transcriptional indicators of signaling pathway activity should be among the genes predominantly controlled by the pathway of interest. We therefore analyzed the response of MPAS genes to MEK/ERK activity modulation in PEO4 cells in vitro (Figure 1). We chose PEO4 cells as a primary experimental model because they display moderate MEK/ERK activity at baseline, and we obtained data about some of their MEK/ERK-related aspects in our earlier report [18].

Fibroblast growth factor 4 (FGF4) binds to its receptors on the cell membrane and stimulates the activation of various signaling pathways, including the MEK/ERK pathway [30]. As expected, the treatment of cells with FGF4 resulted in prominent increases in the phosphorylation of MEK1/2, ERK1/2, and their downstream target p90RSK1 within 30 min after treatment started (Figure 1A). Changes in the expression patterns of MPAS genes in FGF4-treated cells were diverse. *PHLDA1* and *SPRY4* were strongly upregulated (up to a 5- to 6-fold increase after 6 h); *DUSP6*, *EPHA2*, and *ETV5* levels increased 2-fold; and *SPRY2*, *DUSP4*, *ETV4*, and *CCND1* displayed no substantial changes (Figure 1B, Figure S1). As in our previous report [18], *EPHA4* levels in both the control and treated PEO4 cells were below the limit of reliable detection with RT-qPCR, so we excluded this gene from further analysis. *PHLDA1*, *SPRY4*, *EPHA2*, and *ETV5* genes responded to MEK/ERK activation relatively quickly, as their levels were upregulated 2-fold or higher within 2 h after treatment began, while *DUSP6* reached a threshold (2-fold expression increase) after 6 h (Figure 1B, Figure S1). The changes described above are summarized in Table S1.

MEK/ERK pathway activity in tumors, including HGSOC, is tightly connected to cell proliferation and is required for G1-to-S-phase transition in the cell cycle [18]. We therefore hypothesized that changes in MEK/ERK activity and MPAS gene expression during cell cycle progression may be used as a more physiologically relevant model. We used aphidicolin treatment with subsequent release to synchronize PEO4 cells and enriched them in different phases of the cell cycle (Figure 1C, Figure S2). The ERK1/2 activity was strongly elevated during early and mid-S phase (0–3 h timepoints) and then slowly returned to original levels while the cells progressed through G2 and M phases (Figure 1D). Similar to the FGF4 experiment, we could separate the MPAS genes into three groups based on their response to ERK1/2 changes: strong response (*PHLDA1*, *DUSP4*), moderate response (*EPHA2*, *SPRY2*, and *DUSP6*), and weak/no response (*SPRY4*, *ETV4*, *ETV5*, and *CCND1*) (Figure 1E, Figure S3). *PHLDA1*, *DUSP4*, *EPHA2*, and *SPRY2* genes exhibited lasting changes, still displaying more than 2-fold increases in their expression levels 27 h past the release from S phase arrest (Figure 1E, Figure S3; see Table S1 for the summary).

To select the best MEK/ERK responder genes, we assigned points to each evaluated gene based on its performance in the experiments described above and in our previous report (the excerpts of experimental data from our previous study are presented in Figure S4) [18]. The final score obtained allowed us to identify the most reliable responders to MEK/ERK signaling changes (Table 1). Most genes we examined responded robustly to MEK inhibition both in vitro and in vivo, but only some of them displayed reproducible upregulation upon MEK/ERK pathway activation across different models. We therefore concluded that the whole MPAS gene signature exhibited clear redundancy in HGSOC and focused our further analysis on four genes displaying the most robust response to MEK/ERK activity modulation—*PHLDA1*, *SPRY4*, *EPHA2*, and *DUSP4*.

### 2.2. PHLDA1, EPHA2, and DUSP4 Are Associated with Baseline MEK/ERK Activity across Multiple Ovarian Cancer Cell Lines

A prominent gene expression response to various external or internal stimuli is useful for tracking pathway modulation in experimental models. On the other hand, gene expression patterns in clinical samples usually represent either a baseline state or long-term processes. Thus, gene signatures that reflect baseline levels of pathway activity have much higher potential for clinical applicability.

**Figure 2:**
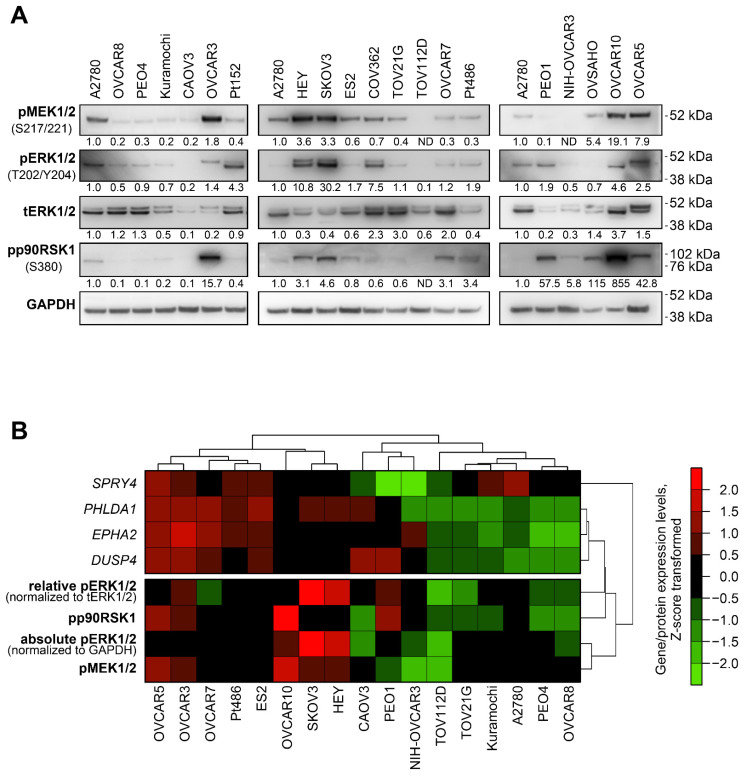
Baseline MEK/ERK pathway activity and PHLDA1, DUSP4, EPHA2, and SPRY4 expression levels in ovarian cancer cell lines. (**A**) Immunoblotting analysis of phosphorylation of MEK/ERK pathway components in 20 human ovarian cancer cell lines. The image represents data obtained from several separate membranes. An A2780 sample was used in each membrane as a reference sample. Numbers under the bands represent relative intensity normalized to GAPDH levels and the A2780 sample. (**B**) Cluster analysis of associations between baseline expression levels of MEK/ERK responder genes and levels of phosphorylated proteins involved in MEK/ERK pathway. Seventeen cell lines displaying consistent activity of several MEK/ERK pathway elements were analyzed. Gene expression levels estimated via RT-qPCR and protein levels estimated via immunoblotting were subjected to Z-score transformation. Unsupervised hierarchical clustering was performed using 1-minus Spearman’s rank correlation metrics.

We evaluated possible associations between the expression of *PHLDA1*, *SPRY4*, *EPHA2*, and *DUSP4* and MEK/ERK pathway activity in 20 ovarian cancer cell lines (Figure 2). The phosphorylation levels of MEK1/2, ERK1/2, and p90RSK1 in these cells were evaluated using immunoblotting. Three cell lines (Pt152, COV362, and OVSAHO) were excluded from further analysis based on discrepancies between individual elements of the MEK/ERK pathway (e.g., high pERK1/2 without pMEK1/2 or high pp90RSK1 with no pERK1/2) (Figure 2A). Individual cell lines displayed marked differences in both protein-estimated MEK/ERK pathway activity and expression levels of MEK/ERK responder genes (Figure 2, Figure S5). We therefore analyzed possible associations between the expression levels of MEK/ERK responders, absolute levels of phosphorylated ERK1/2, MEK1/2, and p90RSK1, and relative levels of pERK1/2 normalized to total ERK1/2 expression, using unsupervised hierarchical clustering and Spearman’s correlation approaches. Three genes (*PHLDA1*, *DUSP4*, and *EPHA2*) displayed positive associations with ERK1/2 phosphorylation levels in both tests, while *SPRY4* exhibited no statistically significant association (Figure 2B, Table 2). We therefore combined *PHLDA1*, *DUSP4*, and *EPHA2* genes into an optimized signature henceforth referred to as “Carcinoma of the Ovary MEK/ERK Signature” (COMS).

### 2.3. COMS Genes Are Suitable for Monitoring Long-Term, but Not Short-Term Transient Changes in MEK/ERK Activity

**Figure 3:**
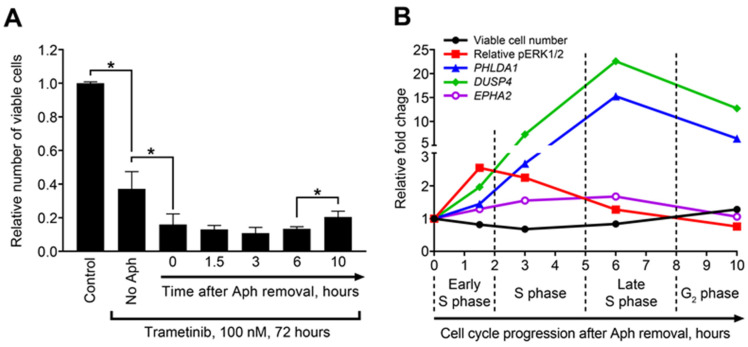
Temporal dynamics of sensitivity to MEK inhibition, ERK1/2 phosphorylation, and MEK/ERK responders’ expression during cell cycle progression. (**A**) Numbers of viable PEO4 cells enriched in different cell cycle phases after subsequent treatment with trametinib. Data are normalized to control samples and presented as mean ± SD. n = 3. * *p* < 0.05, Kruskal–Wallis H test). Aph—aphidicolin. (**B**) Dynamics of cell number reduction (after trametinib treatment), ERK1/2 phosphorylation (prior to trametinib addition), and COMS genes expression (prior to trametinib addition) across different stages of cell cycle. Data are normalized to “0 h” samples and presented as mean values. Relative pERK1/2 levels were calculated by normalization to total ERK1/2 levels.

The level of MEK/ERK pathway activity (defined either via direct protein assessment or biomarker signatures) may define the response of cancer cells to MEK inhibition [26,29]. We therefore tested whether the modulation of MEK/ERK activity and COMS genes during cell cycle progression is associated with the effects of trametinib on cell proliferation. PEO4 cells were enriched in different phases of the cell cycle and subsequently treated with 100 nM trametinib (the lowest concentration providing complete inhibition of MEK/ERK pathway [18]) for 72 h. Cells displaying high pERK1/2 levels (Figure 1C,D) at the moment of trametinib administration demonstrated significantly stronger suppression of their growth (Figure 3A). While phosphorylated ERK1/2 levels exhibited strong negative correlations with the number of viable cells after trametinib treatment, we observed no significant correlation between the expression levels of COMS genes and either pERK1/2 levels or cell number changes (Table S2).

To examine more complex connections between these parameters, we compared the kinetic curves of sensitivity to trametinib treatment and pERK1/2, *PHLDA1*, *DUSP4*, and *EPHA2* levels during the course of cell cycle progression (Figure 3B). As described above, the sensitivity to trametinib peaked within 1.5–3 h after aphidicolin release (early and mid-S phase), when the cells displayed highest pERK1/2 levels prior to trametinib administration. Both effects then gradually decreased. In contrast, the upregulation of COMS genes peaked at 6 h and the *PHLDA1* and *DUSP4* levels remained high even at the 10 h timepoint. Due to such a postponed response, we concluded that COMS responder genes are more suitable for estimating lasting activation of the MEK/ERK pathway (due to either prolonged external stimulation or long-term internal effects) than for monitoring rapid transient modulations in MEK/ERK activity.

### 2.4. COMS Genes Are Overexpressed in HGSOC Samples with High pERK1/2 Levels

**Figure 4:**
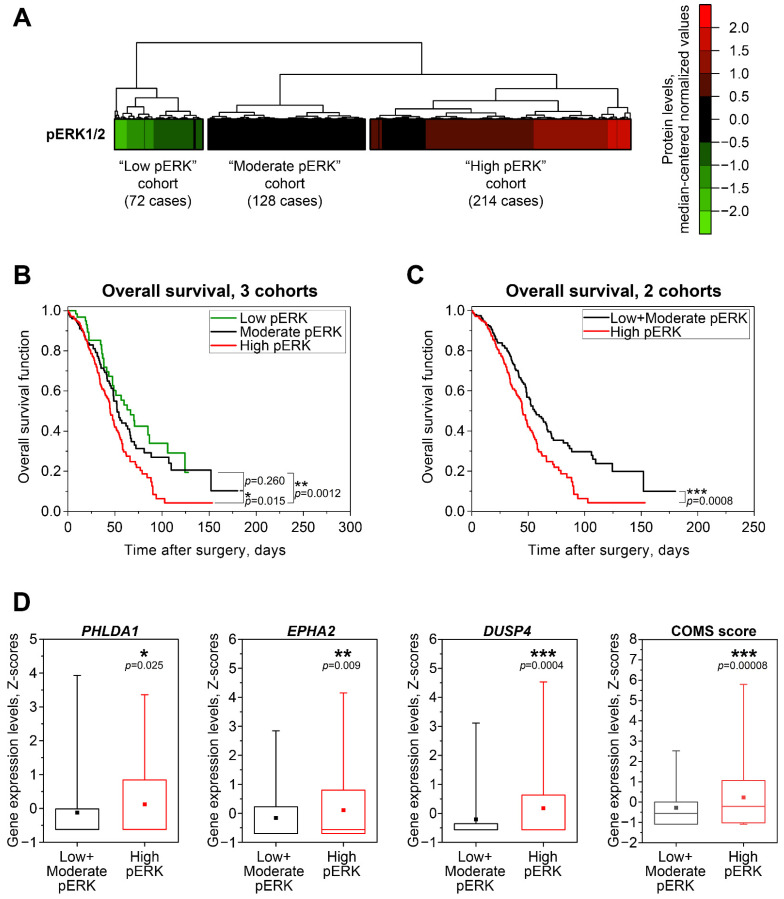
Associations between MEK/ERK pathway activity, patient survival, and MEK/ERK responder genes’ expression in HGSOC patient samples from TCGA. (**A**) Cluster analysis of phosphorylated ERK1/2 protein levels in samples from TCGA “PanCancer Atlas” dataset (n = 414). Unsupervised hierarchical clustering was performed using Euclidean distance metrics. (**B**) Kaplan–Meyer analysis of overall patient survival differences between three pERK1/2-defined cohorts from TCGA “PanCancer Atlas” dataset (n = 414). Statistical significance was evaluated using log-rank tests. (**C**) Kaplan–Meyer analysis of overall patient survival differences between the “High pERK” cohort and combined “Low pERK”+”Moderate pERK” cohort from the TCGA “PanCancer Atlas” dataset. Statistical significance was evaluated using log-rank test. (**D**) Expression levels of COMS genes in samples from the TCGA “Nature 2011” dataset with available pERK1/2 protein data (n = 337). Boxes represent median and quartile values, whiskers represent minimum and maximum values, and squares represent sample mean values. Statistical significance was evaluated using two-tailed T-test with Welch’s correction.

To evaluate the potential of COMS genes to predict baseline MEK/ERK activity in clinical samples, we used proteomic and transcriptomic data for large sets of HGSOC cases available from the “Nature 2011” [16] and “PanCancer Atlas” [31] datasets in The Cancer Genome Atlas (TCGA). First, we analyzed 414 samples with RPPA-based proteomics data using an unsupervised hierarchical clustering algorithm and identified three cohorts of patients exhibiting low (n = 72), moderate (n = 128), and high (n = 214) levels of pERK1/2 protein (Figure 4A). Kaplan–Meyer analysis revealed that low pERK and moderate pERK cohorts displayed no significant differences, while the high pERK group had worse overall survival prognosis (Figure 4B). This difference was even more prominent when we merged the low and moderate pERK cohorts into one group (Figure 4C).

Transcriptomic microarray-based gene expression values are available in the “Nature 2011” dataset for 337 cases of 414 “PanCancer Atlas” cases with proteomic data. High pERK samples exhibited statistically higher expression of *PHLDA1*, *DUSP4*, and *EPHA2* compared to samples from the Low + Moderate pERK cohort (Figure 4D). *SPRY4* expression patterns followed a similar trend, but the differences between cohorts did not reach statistical significance, confirming our previous statement about the inferior potential of *SPRY4* as a marker of MEK/ERK response and baseline activity (Figure S6). We next combined the Z-score values for COMS genes into “COMS score” in the same way as “MPAS score” was calculated [29]. The evaluation of “COMS score” and “MPAS score” indices in the same patient cohorts demonstrated that COMS displays higher statistical power than the single genes (Figure 4D) or full MPAS gene combination (Figure S6). We therefore concluded that estimating the expression of COMS genes may be used as an additional approach for the more reliable evaluation of MEK/ERK pathway functional activity in clinical samples of HGSOC.

### 2.5. COMS Genes Do Not Display Potential to Predict Patient Survival

Since high pERK levels in the HGSOC samples were associated with poor overall survival prognosis, and *PHLDA1*, *DUSP4*, and *EPHA2* were overexpressed in High pERK cases, we evaluated the prognostic potential of these genes and other available clinical parameters using the Cox regression model (Table 3). Neither single COMS genes nor COMS and MPAS signatures displayed significant associations with overall patient survival in a univariate Cox analysis. Initial platinum resistance was identified as a major negative predictor of overall survival, with residual disease and advanced patient age at diagnosis being significant, but minor negative factors. Multivariate Cox regression analysis confirmed the independent prognostic potential of platinum resistance status and demonstrated that residual tumor volume has no independent prognostic value when it is analyzed together with other characteristics. Based on these results, we conclude that the predictive potential of well-established risk factors (advanced age and platinum resistance) for HGSOC patients is superior to examined MEK/ERK responder genes or gene signatures.

## 3. Discussion

MEK/ERK pathway activation is one of the most common molecular events occurring in cancer cells (including, but not limited to, ovarian cancer) and is directly connected to malignant cell proliferation and survival [12,18]. While MEK/ERK signaling is often assessed via the immunochemical detection of ERK phosphorylation, accumulating evidence suggests that this approach may not always correctly represent MEK/ERK pathway activity [25,26]. The detection of transcripts regulated by pathways of interest is a viable alternative due to the high sensitivity and specificity of PCR- and sequencing-based methods. Several transcriptional signatures associated with the functional activity of the MEK/ERK pathway in various tissues were reported in the literature [17,26,27,28,29], and a 10-gene “MPAS” signature displayed a highly robust MEK inhibition response across multiple tumor types [29]. We hypothesized that MPAS signature may be further optimized for ovarian cancer cases and performed detailed evaluations of the expression of MPAS genes in several experimental models and sets of clinical samples of ovarian cancer. Only three out of ten MPAS genes (*PHLDA1*, *EPHA2*, and *DUSP4*) displayed reproducible associations with baseline levels and changes in MEK/ERK activity across most ovarian cancer model systems and clinical samples. Based on these data, we conclude that the MPAS signature is significantly impacted by tissue specificity, model specificity, and the temporal and technical details of experiments.

The identification of universal molecular signatures associated with pathological processes is a goal in biomarker research. Prominent tissue heterogeneity affects the functional properties of even the most ubiquitous genes, and therefore should be considered when evaluating any biomarker signature. Genes are usually considered tissue-specific if they are only expressed in selected tissues due to unique combinations of genetic, epigenetic, and protein-related factors [32,33], but such genes are rarely included in universal signatures. On the other hand, factors involved in the tissue-specific loss of gene expression, including gene silencers [34], alternative transcript variants [35,36], and transcriptional repression [37] may drastically impact the performance of such signatures by rendering their important components unusable. We observed such a situation with the *EPHA4* gene, which was expressed below reliable detection levels in all examined ovarian cancer cell lines. According to the original MPAS report, *EPHA4* contributes significantly to MPAS in lung cancer and melanoma, but is virtually without effect in colorectal cancer, confirming its tissue-specific nature [29]. We therefore suggest that MPAS or any other signature characterized in non-ovarian samples should be verified based on specific patterns of gene expression in ovarian cancer. This statement is further supported by the idea of assigning tissue-specific weights to commonly used gene sets to improve the statistical power of gene set enrichment analyses [38].

While all of our data were obtained from ovarian cancer samples, we utilized several experimental models to investigate the different aspects of MEK/ERK pathway activity changes. On one hand, we performed activation or inhibition of MEK/ERK pathway activity with FGF4 or trametinib treatment, respectively—these model systems reproduce prolonged action of external factors that cause long-term shifts in signaling regulation. On the other hand, we evaluated the temporal dynamics of MEK/ERK activity during cell cycle progression—these recapitulate more short-term, intricate, and physiologically relevant regulatory changes. Lastly, we investigated stable differences in baseline MEK/ERK activity induced by development of platinum resistance, representing lasting changes that are still present after the initial stimulus (cisplatin treatment) is withdrawn.

We observed significant variability between the different experimental models used. *CCND1*, *ETV4*, and *ETV5* responded prominently to pharmacologic MEK/ERK inhibition in cell cultures, but their changes in trametinib-treated HGSOC murine xenografts or different cell line models of MEK/ERK activation were much weaker or even completely absent. On the other hand, *DUSP6* displayed a moderate response to MEK/ERK activity modulations in all tested experimental models except trametinib-treated cells in vitro (Table 1). Such effects, known as model specificity, can be divided into two groups based on the unique effects of: (1) the same stimulus observed in different cells/tissues/animals and (2) similar stimuli observed in the same in vitro/ex vivo/in vivo model system. Both aspects should be considered when analyzing the impact of supposedly similar treatments (e.g., drugs that share a common structure or target the same molecule) performed in different systems (cell lines, organoids, tissue explants, or animals). For example, the treatment of colorectal cancer cells with thalidomide and its derivatives pomalidomide and lenalidomide results in markedly different transcriptomic changes in vitro and in vivo [39]. Moreover, while the tested agents were expected to act in a similar way, Gene Ontology analysis revealed no common categories affected by all three drugs in either in vitro or in vivo models, indicating clear differences in their mechanism of action [39]. Another recent study compared the gene expression response to the proteasome inhibitors ixazomib and bortezomib in human myeloma cell lines and primary patient-derived cells [40]. While in vitro and ex vivo ixazomib treatment models shared more than 200 commonly affected genes, further comparison to effects caused by bortezomib treatment identified only 10 common genes, although both agents selectively target the same 20S proteasome [40,41]. Due to such model specificity, multiple different gene signatures were described for the same drugs or regulatory pathways, resulting in redundancy and decreased reliability [42]. In our comparison of five different experimental models of ovarian cancer, only 4 of 10 MPAS genes displayed consistent and uniform response to MEK/ERK activity modulations, and only 3 (*PHLDA1*, *EPHA2*, and *DUSP4*) were significantly associated with baseline ERK1/2 activity in both cell lines and patient tissue samples, thereby emphasizing the need to validate gene expression signatures for each type of pathology or condition.

The applicability of biomarker signatures for the evaluation of both baseline levels of pathway activity and changes during therapy or disease progression greatly improves their usefulness. While many reported signatures are derived from tumor samples obtained prior to treatment, the signatures based on treatment-induced changes in gene expression displayed superior prognostic potential, most likely due to the association of such signatures with acquired aggressiveness and resistance [43,44].

Platinum resistance is an important prognostic factor for HGSOC patients, and its development is tightly associated with MEK/ERK pathway hyperactivation, which also manifests itself in the overexpression of COMS genes [18,45]. On the other hand, COMS genes displayed no associations with survival among HGSOC patients (Table 3). This fact may be explained by the nature of TCGA samples used in our study, since “Nature 2011” and “PanCancer Atlas” datasets are almost exclusively composed of primary HGSOC cases not treated before sample acquisition. We therefore hypothesize that the evaluation of MEK/ERK responder genes may be more useful in recurrent ovarian tumors for choosing optimal second-line treatment regimens. Moreover, assessing the dynamic changes of MEK/ERK responders may be theoretically used for monitoring the patient response to treatment. Current PCR-based methods are sensitive enough to reliably estimate gene expression levels using extremely low amounts of material, e.g., isolated circulating tumor cells (CTCs), which are detected in 90% of epithelial ovarian cancer patients [46]. The evaluation of COMS genes expression in CTCs may provide a non-invasive way of in vivo tracking of drug efficiency, disease progression, and chemoresistance development.

Increase in MEK/ERK activity (and expression levels of COMS genes) observed in cisplatin-resistant cells was associated with increased sensitivity to MEK inhibition [18], which is consistent with the increased impact of trametinib upon ERK^high^ cells enriched in the S-phase of the cell cycle (Figure 3). Adding MEK/ERK inhibitors to various chemotherapeutic agents [47], including ALDH1A inhibitor [18] and Src inhibitor [22], has been suggested as a way to overcome ovarian cancer therapy resistance. What is even more important is that a number of recent studies reported that MEK inhibition therapy may sensitize ovarian cancer cells to the cytotoxic action of platinum drugs both in vitro and in vivo [45,48,49]. We did not observe significant associations between COMS genes expression and initial platinum sensitivity in clinical cases from TCGA. Nevertheless, we suppose that HGSOC patients with high MEK/ERK activity and COMS genes expression in tumor tissue may benefit from MEK1/2 inhibition, as it may prevent platinum resistance development in the course of initial treatment. Earlier, we reported that trametinib treatment suppresses ERK1/2 phosphorylation and the expression of MEK/ERK-responding genes in ovarian cancer xenografts [18]; it is possible that the efficiency of MEK/ERK-targeting therapy in ovarian and other cancers may be monitored via the detection of COMS genes (or other optimized transcriptional signatures) in fine-needle aspiration biopsy or CTC samples.

Considering the strong association between ERK1/2 phosphorylation modulation and the trametinib sensitivity changes observed during cell cycle progression, it may seem counterintuitive that COMS genes display no significant correlations with either parameter (Table S2). However, our experimental model demonstrates that changes in transcript levels are delayed in comparison to protein changes: the peak level of ERK1/2 phosphorylation level is achieved at 1.5 h after trametinib administration, while gene expression levels reach their maximum values 4.5 h later (Figure 3B). This delay most likely arises from the inherent difference between protein-to-protein signaling (phosphorylation, cleavage, etc.) and the regulation of gene transcription activity. The former is relatively fast as it does not require the synthesis of new molecules, while the latter takes more time because of transcription complex assembly, mRNA synthesis, and transcript processing. On the other hand, the magnitude of the gene expression response may be much higher than the magnitude of the protein changes (*PHLDA1* and *DUSP4* in Figure 3B) due to the accumulation of newly synthesized transcripts over time. Transcript accumulation is strongly connected to lasting response to stimuli (see Table S1), which manifests in the detection of significantly elevated transcript levels long after the MEK/ERK pathway activity returns to baseline levels (Figure 1D,E, Figure S3). Our results suggest that the most potent MEK/ERK responders have a lasting response that is useful for detecting long-term changes in pathway activity, especially in terms of MEK/ERK activation. Due to the same reason, the potential of COMS genes for tracking short-term MEK/ERK activity modulations is limited, since they cannot demonstrate rapid downregulation upon decreased ERK1/2 activity. Several MEK/ERK-regulated genes termed “immediate early response genes” (*FOS*, *BTG2*, and *KLF4*) display prominent, but transient, upregulation within 1–2 h after pathway activation [50]. These genes may be a better choice for monitoring short-term MEK/ERK activity fluctuations, but their expression levels decline over time even with constitutive MEK/ERK pathway activation, rendering their usefulness for possible clinical applications questionable.

## 4. Materials and Methods

### 4.1. Cell Cultures

We used 20 different human ovarian cancer cell lines, including Type I and Type II ovarian cancer cells (see Table S3 for details). The OVCAR8, PEO4, and A2780 cells were provided by Dr. S. Murphy (Duke University, Durham, NC, USA). The OVCAR3, OVCAR5, OVCAR7, OVCAR10, OVSAHO, and ES2 cell lines were provided by Dr. V. Shridhar (Mayo Clinic, Rochester, MN, USA). The Pt152 and Pt486 cells were provided by Dr. R. J. Buckanovich (University of Pittsburgh, Pittsburgh, PA, USA). The Kuramochi cell line was purchased from the Japanese Collection of Research Bioresources Cell Bank (Osaka, Japan). The CAOV3, SKOV3, NIH-OVCAR3, TOV112D (also known as TOV21D), HEY, and TOV21G cells were purchased from the American Type Culture Collection (ATCC, Manassas, VA, USA). The COV362 and PEO1 cells were purchased from the European Collection of Authenticated Cell Cultures (Millipore Sigma, Burlington, MA, USA).

All cell lines were cultivated in RPMI-1640 medium (Corning, Tewksbury, MA, USA) supplemented with 5% FBS (Thermo Fisher Scientific, Waltham, MA, USA) and 100 U/mL penicillin/100 μg/mL streptomycin (Corning, Tewksbury, MA, USA). The PEO1 cells were cultivated in medium with the addition of 1 mM sodium pyruvate (Corning, Tewksbury, MA, USA). The cells were tested for mycoplasma monthly.

### 4.2. Drug Treatment of Cultured Cells

FGF4 (STEMCELL Technologies, Vancouver, Canada) was dissolved in sterile water. Aphidicolin (Cayman Chemical, Ann Arbor, MI, USA) and trametinib (Selleck Chemicals, Houston, TX, USA) were dissolved in DMSO (Fisher Scientific, Waltham, MA, USA). The cells were seeded to the culture dishes and allowed to adhere to the surface overnight. The cell growth media were aspirated and replaced with treatment media containing the drug of interest. The control samples in all experiments performed were treated with vehicle only. The vehicle concentration in growth medium did not exceed 0.2%.

### 4.3. Synchronization of Cultured Cells in Different Phases of Cell Cycle

The cells were cultured in FBS-free growth medium for 24 h and subsequently treated with 2 μg/mL aphidicolin in full growth medium for 20 h to induce cell cycle arrest at the early S-phase. Aphidicolin-treated cells were washed twice with sterile DPBS (Corning, Tewksbury, MA, USA) and either harvested immediately or cultivated in full growth medium for 1.5, 3, 6, 10, or 27 h before harvesting.

### 4.4. Cell Cycle Assays

The cells were harvested by trypsinization, washed once with ice-cold full growth medium, and centrifuged at 200× *g* for 5 min. The cells were resuspended in 300 μL of ice-cold PBS and fixed by the addition of 700 μL of ice-cold 70% ethanol in a dropwise manner with constant mild vortexing. Fixed samples were incubated at −20 °C overnight. The fixed cell samples were washed with ice-cold PBS (Genesee Scientific, El Cajon, CA, USA) twice, treated with 0.2 mg/mL RNAse A (Thermo Fisher Scientific, Waltham, MA, USA) in PBS for 60 min at 37 °C, and stained with 10 µg/mL propidium iodide (Millipore Sigma, Burlington, MA, USA) in PBS. The stained samples were analyzed using an FACSCalibur flow cytometer (BD Biosciences, San Jose, CA, USA) and ModFit LT software (Verity Software House, Topsham, ME, USA).

### 4.5. RT-qPCR Analysis of Gene Expression

The cells were harvested by trypsinization, washed with ice-cold PBS, and centrifuged at 200× *g* for 5 min. The total RNA was isolated from the cell pellets using TRIzol reagent and a PureLink RNA Mini Kit (Thermo Fisher Scientific, Waltham, MA, USA) with on-column DNAse treatment. A RevertAid RT Reverse Transcription Kit (Thermo Fisher Scientific, Waltham, MA, USA) with a combination of oligo(dT)_18_ and random primers was used to generate cDNA from 1 μg of the total RNA. Quantitative PCR analysis of gene expression was performed in a CFX96 Touch thermocycler (Bio-Rad Laboratories, Hercules, CA, USA) using PowerUp SYBR Green Master Mix (Thermo Fisher Scientific, Waltham, MA, USA) and primers listed in Table S4. A three-step amplification program (15 s at 95 °C, 45 s at 62 °C, 30 s at 72 °C) was run for 40 cycles and reaction specificity was checked by melt curve analysis and agarose gel electrophoresis. The reaction efficiency was evaluated using a standard curve approach and was within 98–102% for all primers. The transcript abundance was estimated using Pfaffl’s approach [51]. *TBP* was used as a housekeeping normalization gene.

### 4.6. Immunoblotting

The cells were harvested by scraping in ice-cold PBS and centrifuging at 200× *g* for 5 min. The total protein extracts were obtained from cell pellets using Pierce RIPA buffer (Thermo Fisher Scientific, Waltham, MA, USA) supplemented with Halt Protease and Phosphatase Inhibitor Cocktail (Thermo Fisher Scientific). Protein concentrations were estimated using a Pierce BCA Protein Assay Kit (Thermo Fisher Scientific). Then, 40 μg of the total protein was separated in Bolt 4–12% Bis-Tris Plus Gels (Thermo Fisher Scientific) and transferred to Hybond P 0.45 PVDF membranes (GE Healthcare, Chicago, IL, USA). The membranes were blocked with 5% BSA (Thermo Fisher Scientific) in Tris-buffered saline with 0.1% Tween-20 (TBST) (Fisher Scientific) and probed overnight at 4 °C with the following primary antibodies diluted in 5% BSA in TBST: pMEK1/2 (Cell Signaling Technology, Danvers, MA, USA, 41G9, 1:1000), pERK1/2 (Cell Signaling Technology, D13.14.4E, 1:1000), total ERK1/2 (Cell Signaling Technology, 137F5, 1:1000), pp90RSK1 (R&D Systems, Minneapolis, MN, USA, 1024A, 1:1000), and GAPDH (ProteinTech, Rosemont, IL, USA, 1E6D9, 1:10,000). Secondary antibodies were HRP-conjugated anti-rabbit IgG (Jackson Immunoresearch, West Grove, PA, USA, 1:10,000) or anti-mouse IgG (Jackson Immunoresearch, 1:10,000) diluted in 5% skim milk (Millipore Sigma, Burlington, MA, USA) in TBST. Protein bands were developed using Luminata Classico or Luminata Forte HRP substrate (Millipore Sigma) and detected using an Amersham Imager 600 (GE Healthcare, Chicago, IL, USA). After the band detection, every membrane was incubated in Restore PLUS Western Blot Stripping Buffer (Thermo Fisher Scientific) for 30 min at room temperature and re-probed. The membranes previously stained for pERK1/2 were re-probed for tERK1/2, and other membranes were re-probed for GAPDH. The densitometric analysis of immunoblot images was performed using Image Lab V 6.1.0 software (Bio-Rad Laboratories, Hercules, CA, USA). The raw band intensity values obtained for proteins of interest were divided to corresponding intensity values of GAPDH bands detected using the same immunoblot membrane. The resulting values were additionally normalized to one of the samples, with the relative band intensity level for this sample being equal to 1.00 (see Figure Legends for detailed descriptions of each immunoblot experiment).

### 4.7. Cell Viability Assays

The cells were plated in 12-well plates (Olympus Plastics, El Cajon, CA, USA) at 50,000 cells/well and synchronized in different phases of the cell cycle as described above. Cells enriched in the cell cycle phase of interest were treated with 100 nM trametinib for 72 h. The treated cells were harvested by trypsinization, pelleted, and resuspended in PBS. The numbers of viable and dead cells were assessed by direct counting using a Countess II automated cell counter (Thermo Fisher Scientific) in the presence of 0.4% Trypan Blue.

### 4.8. Analysis of TCGA Datasets

Two publicly available datasets from the TCGA project (https://www.cancer.gov/tcga (accessed on 10 November 2021)) were used: the “Nature 2011” dataset [16] and the “PanCancer Atlas” dataset [31]. The latest versions of both datasets were accessed through cBioPortal website (https://www.cbioportal.org (accessed on 10 November 2021)). The “PanCancer Atlas” dataset contains the highest number of cases with proteomic data (n = 414) obtained via the RPPA approach that includes data for phosphorylated protein forms. These proteomic data (median-centered normalized values) were utilized for the stratification of the patients into cohorts based on pERK1/2 level. Transcriptomic microarray data (Z-scores) were obtained from the “Nature 2011” dataset that has the most prominent overlap with 414 “PanCancer Atlas” cases with proteomic data. The clinical data were combined from information available in both the “PanCancer Atlas” and “Nature 2011” datasets, aiming to compile the most up-to-date information for each case. The aim of transcriptomic and clinical data analysis was to investigate whether COMS genes are associated with the MEK/ERK pathway activity in vivo and to evaluate the ability of COMS genes to predict patient survival.

### 4.9. Statistics

The statistical tests used for the evaluation of differences between the experimental groups are mentioned in the corresponding Figure Legends. Z-score values were calculated after the logarithmic transformation of the data by subtracting the sample mean values from the individual sample values and then dividing the result to the sample standard deviation. Unsupervised hierarchical cluster analysis was performed using Morpheus software (https://software.broadinstitute.org/morpheus (accessed on 17 January 2022)) with the “furthest neighbor” algorithm and either 1-minus Spearman’s rank correlation or Euclidean distance metrics (mentioned in the corresponding Figure Legends). Correlation analysis was performed in OriginPro 2016 software (OriginLab Corporation, Northampton, MA, USA) using a Spearman’s rank correlation approach. Survival analysis was performed in OriginPro 2016 software (OriginLab Corporation) using either the Kaplan–Meyer estimator model with log-rank test or the Cox proportional hazards model. Graphs were plotted using Prism 8 software (GraphPad Software, San Diego, CA, USA) and OriginPro 2016 software (OriginLab Corporation).

## 5. Conclusions

Only 3 of 10 MPAS genes (*PHLDA1*, *DUSP4*, and *EPHA2*) reflect MEK/ERK pathway activity significantly and consistently across multiple experimental models and clinical tissue samples of ovarian cancer. We therefore term this gene signature “COMS” and propose them as preferred targets for the indirect evaluation of MEK/ERK pathway activity in ovarian cancer. The upregulation of *PHLDA1*, *DUSP4*, and *EPHA2* together with MEK/ERK activity is associated with acquired platinum resistance, a crucial negative prognostic factor, thus emphasizing that transcriptional signatures should be preferentially developed with consideration of both baseline and treatment-induced gene expression patterns. Altogether, we conclude that any transcriptional signature, regardless of its robustness and flexibility, should always be validated and optimized for tissue type, tumor type, and the experimental model of interest due to the prominent tissue and model specificity of gene expression in general.

## Figures and Tables

**Table 1:** Summary of MPAS genes’ response to MEK-ERK pathway activity changes across all analyzed experimental models (including data reported in [18], see also Figure S4). Genes emphasized with bold text display the highest potential for their use as MEK/ERK pathway responders.

Gene	Response to MEK-ERK Activity Changes					Total Score
	Inhibition by Trametinib [18] ^a^		Activation by FGF4 ^b^	Modulation during Cell Cycle Progression ^c^	Activation in Cisplatin-Resistant Cells [18] ^d^	
	*In Vitro*	*In Vivo*				
***PHLDA1***	**2**	**2**	**2**	**2**	**2**	**10**
***SPRY4***	**2**	**2**	**2**	**1**	**0**	**7**
***DUSP4***	**2**	**1**	**0**	**2**	**2**	**7**
***EPHA2***	**2**	**1**	**1**	**1**	**1**	**6**
*DUSP6*	0	2	1	1	1	5
*SPRY2*	2	0	0	1	2	5
*ETV4*	2	1	0	0	0	4
*ETV5*	2	1	1	0	0	4
*CCND1*	2	0	0	0	0	2

^a^ Scores are calculated as follows: more than 2-fold decrease—2 points, 1.5–2-fold decrease—1 point; ^b^ Scores are calculated as follows: strong response—2 points, moderate response—1 point; ^c^ Scores are calculated as follows: strong response—2 points, moderate response—1 point; ^d^ Scores are calculated as follows: more than 4-fold increase—2 points, 2–4-fold increase—1 point.

**Table 2:** Associations between expression levels of the most promising MEK/ERK responders and relative ERK phosphorylation levels in untreated ovarian cancer cell lines. Positive correlation coefficients imply that two parameters of interest display conforming trends (“both high” or “both low”) in the same samples, ranging from “0” (extremely weak association) to “1” (extremely strong association). Values in “0.5–0.6” interval imply a moderate-to-strong association between parameters. *p*-value reflects the statistical significance of correlation coefficient being correctly calculated. Genes emphasized with bold text display statistically significant associations with ERK1/2 phosphorylation.

MEK/ERK-Responding Gene	Correlation Parameters	Correlation to Relative ERK1/2 Phosphorylation Level
***PHLDA1***	**Spearman’s correlation coefficient**	**0.600**
	***p*-value**	**0.014**
***DUSP4***	**Spearman’s correlation coefficient**	**0.588**
	***p*-value**	**0.017**
***EPHA2***	**Spearman’s correlation coefficient**	**0.550**
	***p*-value**	**0.027**
*SPRY4*	Spearman’s correlation coefficient	0.144
	*p*-value	0.594

**Table 3:** Cox proportional hazards analysis of associations between clinical parameters of HGSOC cases, COMS and MPAS gene expression levels, and overall survival of HGSOC patients from TCGA datasets. Hazard ratios higher than “1” indicate that the clinical parameter of interest is associated with poor survival, while hazard ratios less than “1” define parameter of interest as a factor of favorable prognosis. Clinical parameters emphasized with bold text display statistically significant associations with patients’ survival.

Clinical Parameters	Hazard Ratio	*p*-Value
**Univariate Cox Model Estimation**		
**Platinum resistance**	**3.7224**	**<0.0001**
**Tumor residual disease**	**1.2603**	**0.0015**
**Age at diagnosis**	**1.0216**	**0.0064**
Aneuploidy score	1.0183	0.0856
Neoplasm histologic grade	1.3342	0.2017
Fraction of genome altered	0.6311	0.2711
*PHLDA1* expression	1.0599	0.4097
*DUSP4* expression	1.0776	0.3085
*EPHA2* expression	1.0433	0.5708
COMS score	1.0664	0.2662
MPAS score	1.0115	0.7515
Multivariate Cox model estimation		
**Platinum resistance**	**2.9851**	**<0.0001**
**Age at diagnosis**	**1.0277**	**0.0041**
Tumor residual disease	1.0438	0.6766

## Data Availability

Data from the “Nature 2011” and “PanCancer Atlas” datasets used in the present study are available from TCGA Research Network (https://www.cancer.gov/tcga (accessed on 10 November 2021)) through cBioPortal website (https://www.cbioportal.org (accessed on 10 November 2021)) [16,31].

## Supplementary Materials

The following supporting information can be downloaded at: https://www.mdpi.com/article/10.3390/ijms232113365/s1.
