# Supplementary material for: Optimized Transcriptional Signature for Evaluation of MEK/ERK Pathway Baseline Activity and Long-Term Modulations in Ovarian Cancer
Source: Int J Mol Sci. 2022 Nov 1;23(21):13365. doi: 10.3390/ijms232113365 (PMC9654336; doi:10.3390/ijms232113365)
Supplement: Supplementary file 1 [file ijms-23-13365-s001.zip › ijms-1915391-supplementary.pdf]

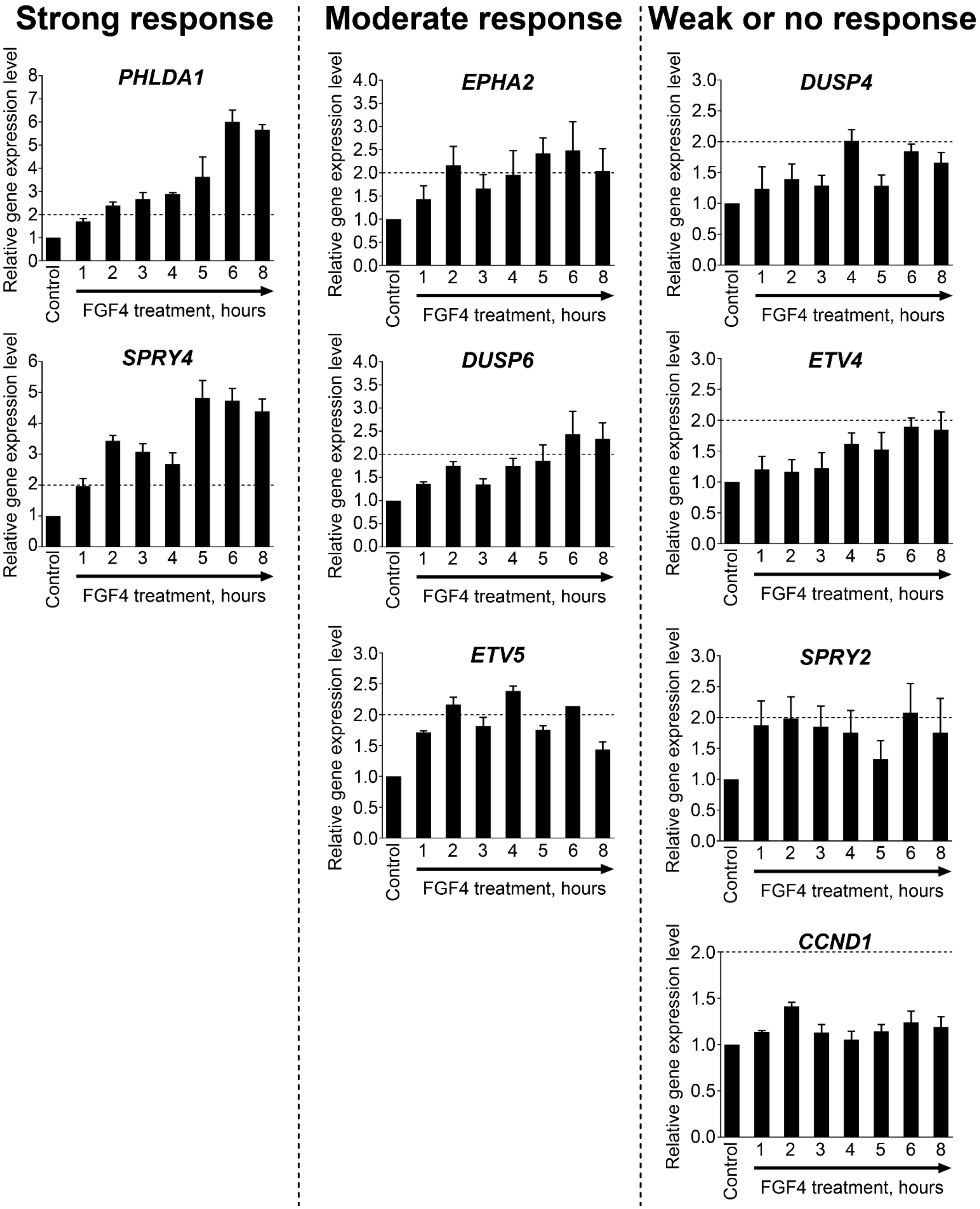

Figure S1. Expression levels of MPAS genes in PEO4 cells treated with FGF4 (100 ng/mL). Data are normalized to "Control" sample and presented as geometric mean + S.E.M. (N=3, technical replicates).

Table S1. Summary of MPAS genes' response to MEK-ERK pathway activation in cell cultures induced by FGF4 treatment or observed during cell cycle progression.

Gene	Response magnitude		Response duration[c]
	FGF4 treatment[a]	Cell cycle progression[b]	
PHLDA1	Strong	Strong	Lasting
EPHA2	Moderate	Moderate	Lasting
DUSP4	Weak	Strong	Lasting
DUSP6	Moderate	Moderate	Transient
SPRY4	Strong	Moderate	Transient
SPRY2	Weak	Moderate	Lasting
ETV5	Moderate	Weak	Transient
ETV4	Weak	Weak	Transient
CCND1	No Response	No Response	N/A

[a]Magnitude of gene expression changes in experiment with FGF4 treatment:

- Strong – more than 4-fold increase;
- Moderate – 2-4-fold increase;
- Weak – 1.5-2-fold increase;
- No Response – less than 1.5-fold increase.

[b]Magnitude of gene expression changes in experiment with cell cycle progression :

- Strong – more than 10-fold increase;
- Moderate – 2-10-fold increase;
- Weak – 1.5-2-fold increase;
- No Response – less than 1.5-fold increase.

[c]Duration of gene expression changes in experiment with cell cycle progression :

- Lasting – gene expression remains upregulated at the end of the experiment;
- Transient – gene expression returns to baseline level at the end of the experiment;
- N/A – not applicable due to lack of response.

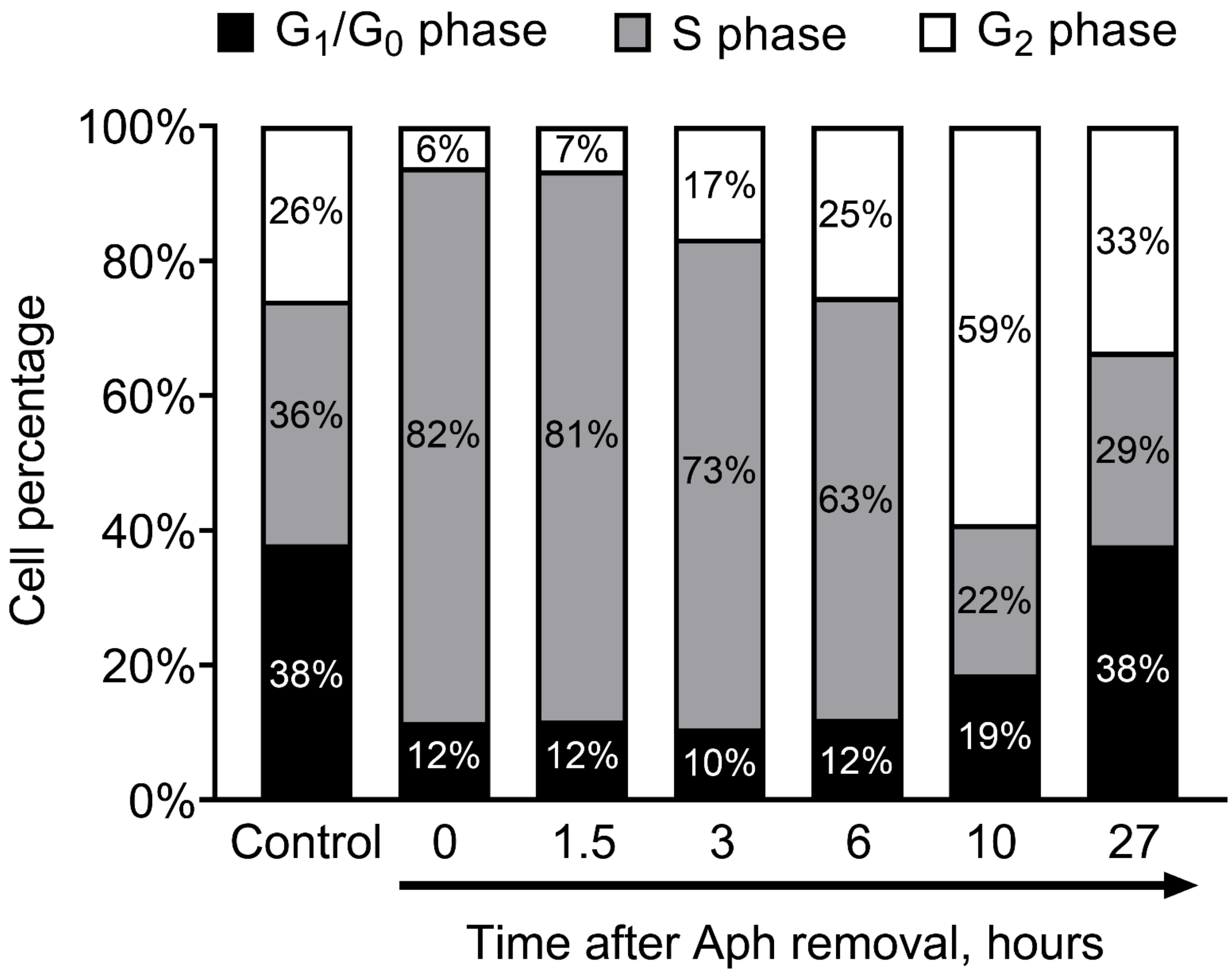

Figure S2. The ratios between cell cycle phases in PEO4 cells synchronized with aphidicolin treatment and subsequently released from it. Aph - aphidicolin.

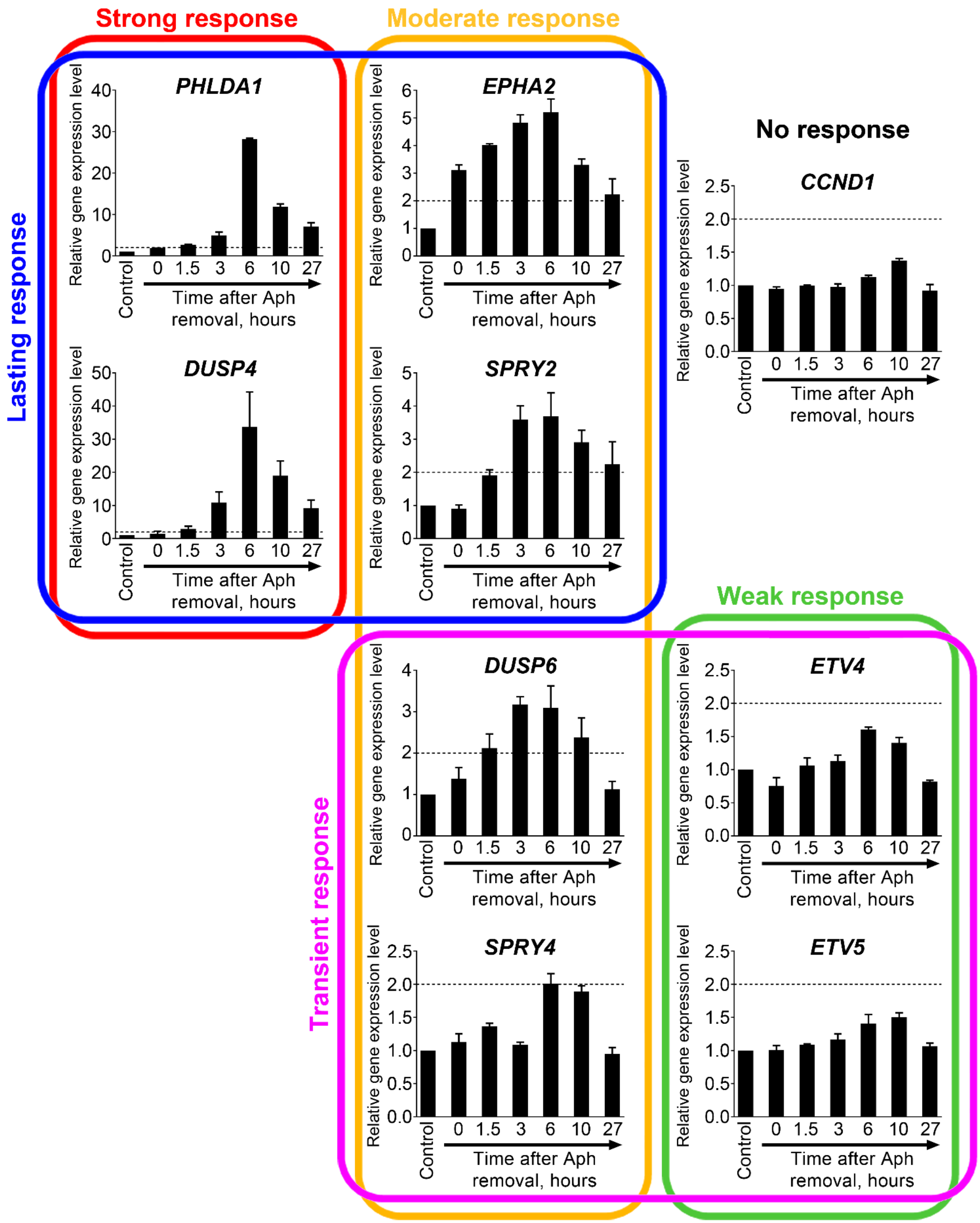

Figure S3. Expression levels of MPAS genes in PEO4 cells enriched in different stages of cell cycle. Data are normalized to "Control" sample and presented as geometric mean + S.E.M. (N=3, technical replicates). Aph – aphidicolin.

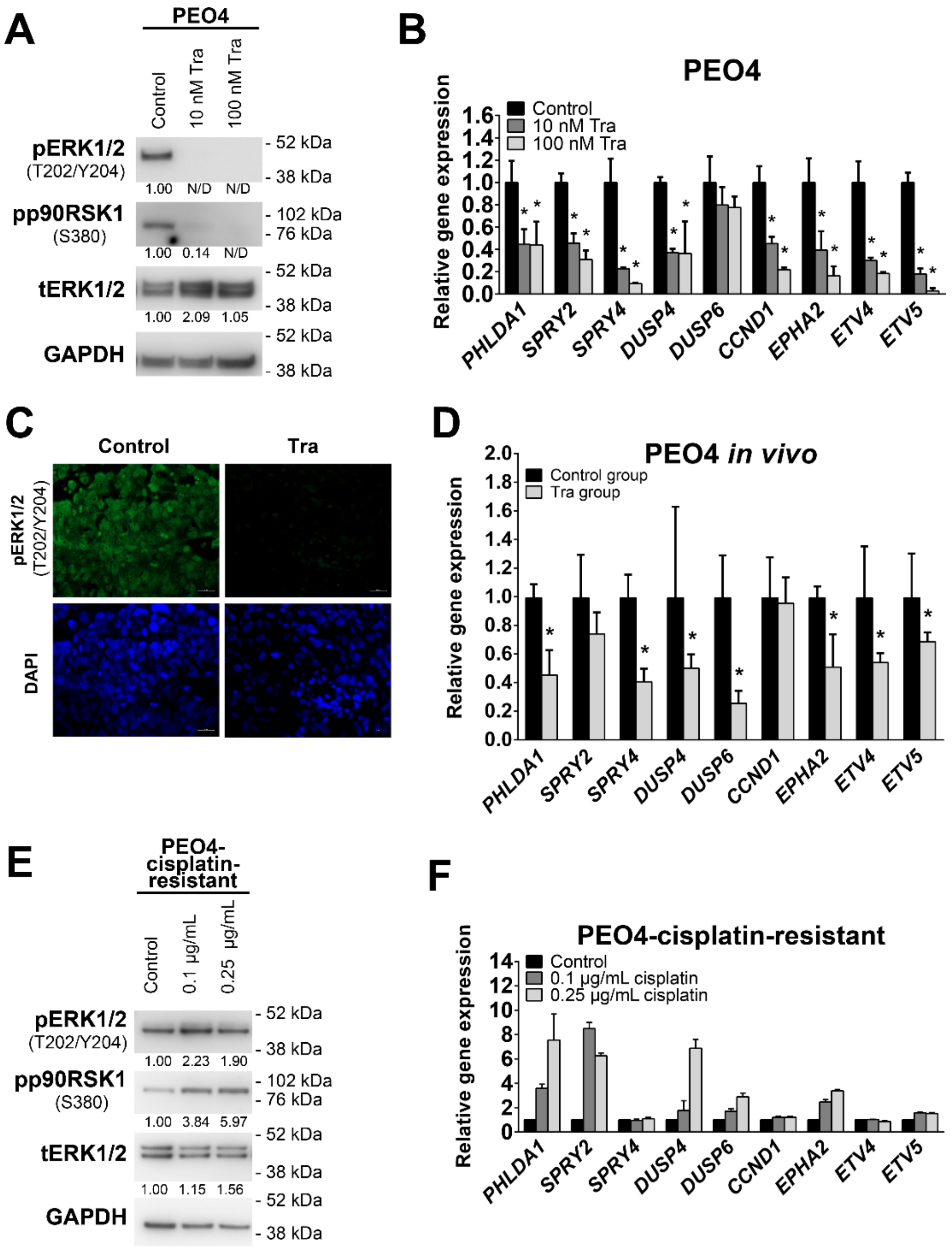

Figure S4. Changes in MEK/ERK pathway activity and expression of MPAS genes observed in PEO4 cells treated with trametinib *in vitro*, PEO4-generated xenografts *in vivo*, and cisplatin-resistant PEO4 cells (the images are reproduced with a permission from Chesnokov et al., 2021 [18]). (A) Immunoblotting analysis of MEK/ERK pathway components activation in cells treated with trametinib for 24 h. Numbers under the bands represent relative intensity normalized to GAPDH levels and Control sample. (B) Gene expression levels of MPAS genes in cells treated with trametinib for 10 h. Data are normalized to "Control" samples and presented as mean+S.D. (N=3, two-tailed Student's T-test with Welch's correction, * $P<0.05$). (C) Immunofluorescent staining of phosphorylated ERK1/2 (green) in xenograft tissue samples. Cell nuclei were counterstained with DAPI (blue). Scale bars: 20 µm. (D) Gene expression levels of MPAS genes in xenograft tissue samples. Data are normalized to "Control group" samples and presented as mean + S.D. (N=3, two-tailed Student's T-test with Welch's correction, * $P<0.05$). (E) Immunoblotting analysis of MEK/ERK pathway component activation in cells resistant to the indicated concentrations of cisplatin. Numbers under the bands represent relative intensity normalized to GAPDH levels and Control samples. (F) Gene expression levels of MPAS genes in cells resistant to the indicated concentrations of cisplatin. Data are normalized to "Control" samples and presented as mean+S.E.M. (N=3, technical replicates). Tra—trametinib, N/D—non-detectable signal.

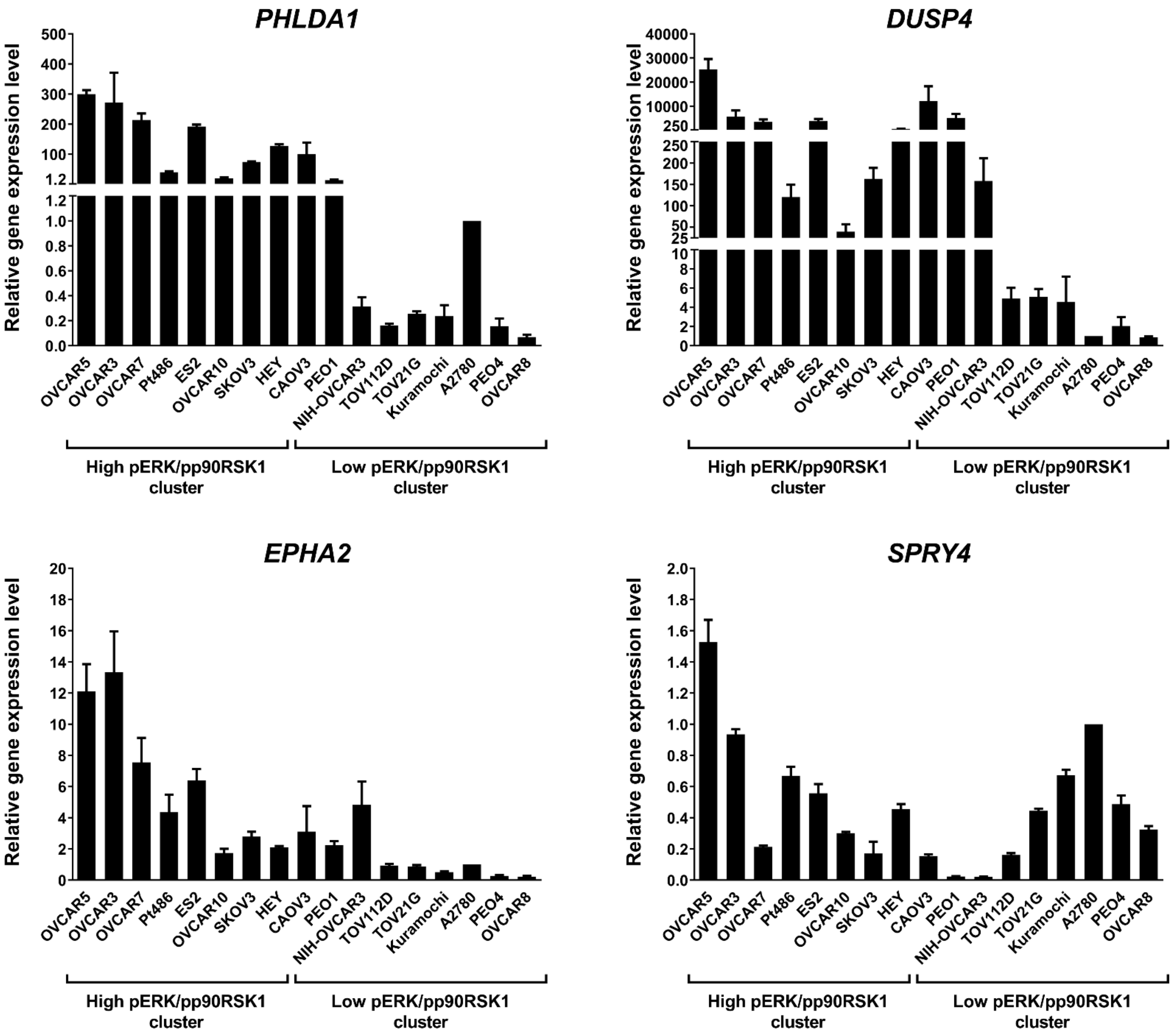

Figure S5. Baseline expression levels of the most robust MEK/ERK responder genes (*PHLDA1*, *DUSP4*, *EPHA2*, and *SPRY4*) in human ovarian cancer cell lines. Data are normalized to "A2780" sample and presented as geometric mean + S.E.M. (N=3, technical replicates). "High pERK/pp90RSK1 cluster" and "High pERK/pp90RSK1 cluster" are determined based on analysis presented in Figure 2B.

Table S2. Spearman's correlation analysis of associations between ERK phosphorylation changes, COMS genes expression changes, and viable cell numbers in PEO4 cells treated with trametinib during different phases of cell cycle. Positive correlation coefficients imply that two parameters of interest display conforming trends ("both high" or "both low") in the same samples. Negative correlation coefficients imply that two parameters of interest display opposite trends ("one is high, another is low") in the same samples. These coefficients range from "0" (extremely weak association) to "1" or "-1" (extremely strong association). Values close to "-1" define a very strong trend of "the higher is ERK1/2 phosphorylation, the lower number of viable cells is left after trametinib treatment" and vice versa. *P* value reflects the statistical significance of correlation coefficient being correctly calculated.

MEK/ERK-responding gene	Correlation parameters	Correlation to viable cell number after trametinib treatment	Correlation to relative ERK1/2 phosphorylation level
Viable cell number after trametinib treatment	Spearman's correlation coefficient	1.000	**-0.943**
	P value	-	**0.005**
Relative ERK1/2 phosphorylation level	Spearman's correlation coefficient	**-0.943**	1.000
	P value	**0.005**	-
PHLDA1 expression level	Spearman's correlation coefficient	-0.371	0.314
	P value	0.468	0.544
DUSP4 expression level	Spearman's correlation coefficient	-0.371	0.314
	P value	0.468	0.544
EPHA2 expression level	Spearman's correlation coefficient	-0.771	0.714
	P value	0.072	0.111

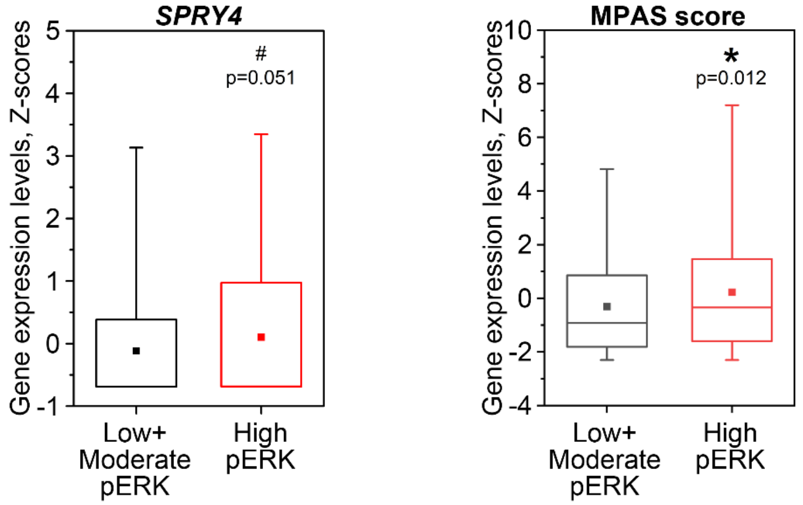

Figure S6. MEK/ERK responder genes' expression in HGSOC patient samples from TCGA. SPRY4 gene expression levels and MPAS scores in samples from the TCGA "Nature 2011" dataset with available pERK1/2 protein data (N=337). Boxes represent median and quartile values, whiskers represent minimum and maximum values, and squares represent sample mean values. Statistical significance was evaluated using two-tailed T-tests with Welch's correction.

Table S3. Human ovarian cancer cell lines used in the study.

Cell line	Ovarian cancer type	Exact tumor form	Additional features
PEO1	Type II	High grade serous adenocarcinoma	Platinum-sensitive
PEO4	Type II	High grade serous adenocarcinoma	Platinum-resistant
Kuramochi	Type II	High grade serous adenocarcinoma	
CAOV3	Type II	High grade serous adenocarcinoma	
OVCAR3	Type II	High grade serous adenocarcinoma	Is often referred to as “NIH-OVCAR3”, but had clear morphological and transcriptional differences from our “NIH-OVCAR3” cells.
OVCAR5	Type II	High grade serous adenocarcinoma	Possibly misclassified, may originate from gastrointestinal tract. $\textit{KRAS}^{G12V}$ mutation.
OVCAR7	Type II	High grade serous adenocarcinoma	
OVCAR8	Type II	High grade serous adenocarcinoma	$\textit{KRAS}^{P121H}$ mutation
OVCAR10	Type II	High grade adenocarcinoma	
Pt152	Type II	High grade serous adenocarcinoma	Derived from primary HGSOC patient cells
NIH-OVCAR3	Type II	High grade serous adenocarcinoma	Is often referred to as “OVCAR3”, but had clear morphological and transcriptional differences from our “OVCAR3” cells.
OVSAHO	Type II	High grade serous adenocarcinoma	
A2780	Type I	Endometrioid carcinoma	
HEY	Type I	Papillary cystadenocarcinoma	$\textit{BRAF}^{G464E}$ and $\textit{KRAS}^{G12D}$ mutations
SKOV3	Type I	Papillary cystadenocarcinoma	
ES2	Type I	Clear cell adenocarcinoma	$\textit{BRAF}^{V600E}$ mutation
COV362	Type I	Endometrioid carcinoma	
TOV21G	Type I	Clear cell adenocarcinoma	$\textit{KRAS}^{G13C}$ mutation
TOV112D	Type I	Endometrioid carcinoma	Is often referred to as “TOV21D”
Pt486	Type I	Undetermined	Derived from primary ovarian cancer patient cells

Table S4. List of primers used in RT-qPCR analysis.

Gene	Forward primer	Reverse primer
TBP	GAGAGTTCTGGGATTGTACC	GGATTATATTCGGCGTTTCG
PHLDA1	GGCAAGACAAGGTTTTGAGG	TCGCAAGTTTTCAGTAGGGTG
SPRY2	TCCACTCAGCACAAACAC	GATTATGCCATCAGCAACAG
SPRY4	CAACGGCTCTTAGACCAC	CACACTCCTTGCATTTACAC
DUSP4	CCCCACTACACGACCAG	TCCGAGGAGACATTCAACAG
DUSP6	GACGCTCGCTGTTTGTATC	GCTTCTAATCCCTCCCTCC
CCND1	CTGGATGCTGGAGGTCTG	GGTCTCCTTCATCTTAGAGGC
EPHA2	GTCAGCATCAACCAGACAGA	TCCCTTCTTGCGGTAAGTG
EPHA4	CGGAGCGGAGAATGC	TCCTTCCCAGACAGAGTAG
ETV4	AGGAGACATCAAGCAGGAAGGG	CCCAGAGCCTGGCGACC
ETV5	AGCACAAGTTCCTGATGATG	CATAGTTAGCACCAAGAGCC
